# Influence of Input Parameters on Dynamic Orbital Stability of Walking: In-Silico and Experimental Evaluation

**DOI:** 10.1371/journal.pone.0080878

**Published:** 2013-11-15

**Authors:** Federico Riva, Maria Cristina Bisi, Rita Stagni

**Affiliations:** 1 Department of Electrical, Electronic, and Information Engineering ‘Guglielmo Marconi’, University of Bologna, Bologna, Italy; 2 Health Sciences and Technologies – Interdepartmental Center for Industrial Research (HST-ICIR), University of Bologna, Bologna, Italy; Cuban Neuroscience Center, Cuba

## Abstract

Many measures aiming to assess the stability of human motion have been proposed in the literature, but still there is no commonly accepted way to define or quantify locomotor stability. Among these measures, orbital stability analysis via Floquet multipliers is still under debate. Some of the controversies concerning the use of this technique could lie in the absence of a standard implementation. The aim of this study was to analyse the influence of i) experimental measurement noise, ii) variables selected for the construction of the state space, and iii) number of analysed cycles on the outputs of orbital stability applied to walking. The analysis was performed on a 2-dimensional 5-link walking model and on a sample of 10 subjects performing long over-ground walks. Noise resulting from stereophotogrammetric and accelerometric measurement systems was simulated in the in-silico analysis. Maximum Floquet multipliers resulted to be affected by both number of analysed strides and state space composition. The effect of experimental noise was found to be slightly more potentially critical when analysing stereophotogrammetric data then when dealing with acceleration data. Experimental and model results were comparable in terms of overall trend, but a difference was found in the influence of the number of analysed cycles.

## Introduction

Stability, in terms of capability to walk without falling or stumbling, is a crucial feature of gait [1,2]. Loss of dynamic stability while walking can lead to falls, which represent a major problem for community and public health, with large clinical and economic consequences [3,4]. Moreover, the majority of fall-related injuries in older adults occur during walking [5–7]. The possibility to detect a loss of stability, offline or in real-time, would represent an improvement in the understanding of the mechanisms related to falls. The capability to quantify decreased dynamic stability could lead to the development of devices alerting the subject (or the clinician) of potentially critical situations in order to prevent the fall, particularly in the case of long walks. Moreover, subjects with low gait stability could be selected for fall prevention programs.

Several stability indices have been proposed in the literature for clinical application [2,7–10], among them, measures coming from nonlinear analysis of dynamical systems are particularly interesting.

Many human tasks are structurally cyclic, and show a periodic-like behaviour. A motor task can be treated as a nonlinear dynamic system: biomechanical variables (e.g. joint angles, accelerations) vary during the temporal evolution of the task, defining a system whose kinematics continuously changes over time according to a controlled pattern. Techniques for nonlinear stability analysis basically consist in the quantification of the tendency of an orbit (defined by the temporal evolution of a set of variables called *state space*) to diverge from (or converge to) the previous orbit or a repelling/attracting limit cycle.

Two main approaches for nonlinear stability analysis in biomechanics are proposed in the literature: local and orbital stability analysis. These nonlinear measures of dynamic stability quantify different properties of system dynamics [11].

In particular, orbital stability analysis can be applied to periodic systems with a limit cycle behaviour; it has been extensively used in the study of passive dynamic walking robots [12] and, in recent years, it has been applied to biomechanics also [2,11,13–16]. Fundamental indicators of orbital stability are Floquet multipliers (FM). FM quantify, discretely from one cycle to the next, the tendency of the system's states to return to the periodic limit cycle orbit. If FM have magnitude < 1, perturbations tend to shrink by the following repetition, and the system remains stable [11]. Smaller FM’s imply higher stability [17].

Despite the lack of evidence of a direct correlation between maxFM and fall risk [10], still FM were found to be higher in fall-prone older adults than in healthy subjects [1], and capable to detect perturbations during walking [18]. For this reason, FM could be used in the detection of real time short-term potentially critical variations in stability.

Deriving from the nonlinear analysis of dynamic systems, orbital stability analysis finds its main application in robotics. When assessing the stability of a robot (e.g. a walker), the equations of motion and the nature of the controllers are known, allowing an adequate selection of the variables that properly characterize the system and the implementation of the analysis in an analytical or semi-analytical way [19]. However, when dealing with human biomechanical time series, equations of motion and control laws are unknown. FM must hence be calculated numerically, and with no a-priori knowledge on the more appropriate variables that define the system. This lack of knowledge makes the implementation not straightforward.

Beyond the mathematical implications, it is however important to highlight that applying this analysis to human gait implies several assumptions: i) human gait is an inherently stochastic system, while Floquet theory applies to deterministic limit cycle systems; ii) walking trajectories are continuously "re-perturbed" by stochastic perturbations that are often internal to the system. Since one of the main assumptions behind the application of this technique is the existence of a limit cycle trajectory, a reference trajectory for human stable walking has to be chosen. To cope with this situation, the average trajectory during the motor task is assumed as limit cycle, although the likely asymmetrical nature of the basin of attraction of human walking. 

Orbital stability analysis preliminarily resulted to detect gait instability [1,18], suggesting its effectiveness despite the many theoretical assumptions, but reference values for orbital stability of stable human walking are not known and, in the literature, incoherent results are reported [20]. This incoherence is likely to result from the absence of a standard implementation of the technique. In particular, the influence to experimental input noise, state space construction, and analysed cycles has not been characterized yet. No unique way of defining the state space of a given motor task was defined in the literature: which and how many variables should be included in the state space and how this choice affects the results of the numerical calculation of orbital stability analysis have not been analysed yet. A similar problem was examined in the literature [21]: the performance of local dynamic stability was analysed when applied to a Lorentz attractor and an experimental sewing task, but this did not allow to draw clear conclusions about locomotion. Another relevant issue is the minimum and optimum number of task cycles to be analysed in order to obtain reliable orbital stability results: this issue was addressed before [22], but only for experimental treadmill walking. Moreover, it is not clear yet how the experimental noise can affect FM calculation.

In this scenario, the analysis of physiological signals of gait (e.g. accelerations, joint angles) from a walking stable model can allow the assessment of the influence of i) experimental noise, ii) state space variables and iii) number of analysed cycles on FM values.

In order to obtain indications applicable in experimental conditions, model data must be comparable with experimental data. Signals extracted from a stable walking model are hence required.

Some authors performed simulation studies on orbital stability of 1 or 2-link walking models related to fall risk [19,23,24]. However, these are simplified models and simulate very peculiar walking conditions. Simplicity is both the strength and the limitation of these models: their walking conditions can be easily manipulated, but they generate signals that significantly differ from physiologic human gait. Stability analysis on a more complex model can better describe human walking, allowing the comparison between model and experimental results. In order for the model to produce kinematics as similar as possible to stable human gait, the required conditions for the model are a continuous walk and the absence of falls or stumbles, regardless of control laws and implementation details.

The aim of the present study was to analyse, from an applicative point of view, the influence on the final results of orbital stability analysis applied to walking of i) experimental measurement noise, ii) selection of the variables for the reconstruction of the state space iii) number of analysed cycles on a 2-dimensional 5-link walking model [25], providing walking patterns of known stability. Results of in-silico analysis were compared to those obtained experimentally on 10 subjects performing long overground walks. 

## Methods

### Overview

In-silico orbital stability analysis of a 5-link stable walking model [25] was performed. The model showed continuous walking, free of falls or stumbles, for all the simulation period (300 strides). This was also assured by a check on step variability, which was minimal following visual inspection of the phase portraits. In order to properly calculate orbital stability, model was slightly perturbed. The analysis was performed for increasing number of cycles (from 10 to 300), based on differently composed state spaces (including different joint angles and/or accelerations). Semi-analytical value of the maxFM of the model was also calculated for reference. Simulated experimental error and noise were added to the segmental kinematics of the model and the sensitivity of orbital stability analysis was evaluated. Orbital stability analysis was also performed on data collected experimentally on 10 subjects; given the impossibility to use a stereophotogrammetry system on a long outdoor road, only acceleration data were acquired experimentally. Orbital stability was calculated using an established technique [13].

### In-silico data

**Figure 1:**
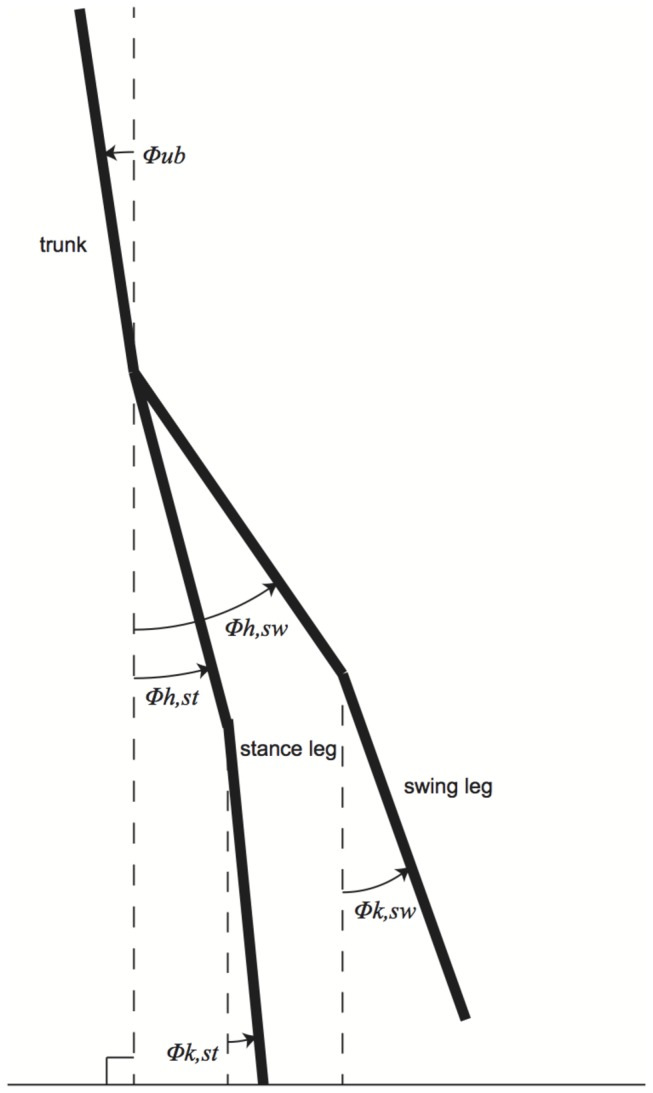
Schematic representation of the 5-link 2-dimensional model (Solomon et al., 2010).

The 2-dimensional, 5-link biped walking model analysed [25] consisted of a trunk, two thigh and two shank segments (Figure 1). The model orientation was described by stance and swing knee angles, stance and swing hip angles and upper body angle (*φ*_*k,sw*_, *φ*_*k,st*_, *φ*_*h,st*_, *φ*_*h,sw*_, *φ*_*ub*_, all referred to gravity direction). The dynamics of the model consisted of a continuous swing phase during which the swing leg smoothly swinged past the stance leg, and an istantaneous, fully inelastic heel strike when the swing foot touched the floor. The swing leg knee contained a hyper-extension stop and a latch that was activated upon full extension to hold the leg straight. There were a total of four control torques on the model, at the stance/swing knee and at the stance/swing hip. All control torques were implemented with fixed gain proportional-derivative (PD) controllers (with *kp* = 100 Nm and *kd* = 10 Nms).

Small random perturbations were added to the state variables at each heel strike event as uniformly distributed random numbers having maximum amplitude ±10*10^-4^. This maximum amplitude was chosen based on the maximum perturbation that the model could tolerate without falling.

The model was adapted to perform 315 consecutive strides. The first 15 strides of the simulation were discarded in order to assure stable walking condition. The simulation was performed using a MATLAB’s (Mathworks, Natwick, NA) fourth- and fifth- order variable time-step Runge-Kutta solver (ode45, with relative error tolerance set to 10^-12^). Joint angles were expressed using Grood and Suntay approach [26]. Accelerations of the trunk segment at the level of the fifth lumbar vertebra (L5) were obtained by the second derivative of the position of a point located at 1/8 of the trunk segment.

Segmental kinematics data obtained from the model were used to reconstruct experimental data from a stereophotogrammetric system (joint angles) and a single inertial sensor located on the trunk (accelerations). Simulated experimental noise and errors were superimposed to segmental kinematics signals obtained from the model.

Clusters of 4 markers were virtually applied to all the segments of the model (trunk, thighs and shanks, for a total of 20 markers) and simulated instrumental normally distributed noise with a standard deviation of 0.2 mm was added to the marker position in 2-d space. Technical reference frames were calculated from the cluster positions, and the position of the segment extremities relative to these frames was estimated. A mis-localization error of anatomical landmark positions (Table 1) was also added to the estimate of the position of segment extremities [27]. Joint angles were then calculated from the relative orientation of the anatomical reference frames [28]. 

**Table 1:** Precision of the palpable anatomical landmark position (in millimeters) in the relevant mean anatomical frame obtained by Della Croce et al., 1999.

Anatomical landmark	*x*	*y*
Greater trochanter (GT)	12.2	11.1
Medial Epicondyle (ME)	5.1	5.0
Lateral Epicondyle (LE)	3.9	4.9
Medial Malleolus (MM)	2.2	2.6
Lateral Malleolus (LM)	2.6	2.4

For ME, LE and MM, LM the mean value between the two was used in the analysis.

Instrumentation noise (white noise with an SNR of 10 dB and alignment errors with a normal distribution and a standard deviation of 0.1 degrees), compatible with use of commercial accelerometers, was added to acceleration signals of the trunk segment at the level of L5. Smaller magnitudes of noise were also analysed that led to comparable results, therefore, only the most potentially critical conditions were reported.

### Experimental data

10 healthy participants [age 28 ± 3 years, height 174 ± 11 cm, weight 67 ± 13 kg] were included in the study. Two synchronized tri-axial inertial sensors (Opal, APDM, Portland, OR, USA) were placed on the participants at the level of L5 and of the right shank, for measuring angular velocity of the lower leg. Accelerations and angular velocities were recorded. The range of the accelerometers was ±2G and sampling rate was 128 samples/second. The participants were instructed to walk straight at self-selected speed on a 250 m dead-end long road.

### Ethics Statement

The Bioetihcal Committee of the University of Bologna approved this study (July 7, 2012). Written informed consent was obtained from the participants.

### Data processing

Orbital stability analysis was implemented according to methodology described in the literature [13,23,24,29].

Seven state spaces (six for model-data and one for experimental data) were analysed (Table 2), based on the literature about orbital stability of human gait [11,15,16]. Two approaches were used. Five state spaces were constructed directly including time series into the state space. These state spaces (Table 2) included model knee flexion-extension joint angles (WMk), model hip flexion-extension joint angles (WMh), model knee+hip+trunk flexion-extension joint angles (WMhkt) and experimental accelerations in the V and AP directions (EXPa). Two state spaces were constructed using delay embedding [30,31] of model AP (WMaAP) and V (WMaV) trunk acceleration signals. An embedding dimension of *dE* = 5 was chosen, as several published studies supported this delay dimensions appropriate for gait data [22,31,32]. A fixed time delay τ = 10 was chosen [22,32].

**Table 2:** Description of the state spaces.

Acronym	Description	Composition
WMk	Swing+stance knee flexion/extension joint angles (model)	*WMk*(*t*) *=* [*ϕ*_*k,st*_(*t*)*, ϕ*_*k,sw*_(*t*)] ∈ R^2^
WMh	Swing+stance hip flexion/extension joint angles (model)	*WMh*(*t*) *=* [*ϕ*_*h,st*_(*t*)*, ϕ*_*h,sw*_(*t*)] ∈ R^2^
WMhkt	Knees, hips and trunk flexion/extension joint angles (model)	*WMhk*(*t*) *=* [*ϕ*_*k,st*_(*t*)*, ϕ*_*k,sw*_(*t*)*, ϕ*_*h,st*_(*t*)*, ϕ*_*h,sw*_(*t*)*, ϕ*_*t*_(*t*)] ∈ R^5^
WMaAP	5-dimensional delay embedding of AP accelerations of L5 (model)	*WMaAP*(*t*) *=* [*a*_*AP*_(*t*)*, a*_*AP*_(*t + τ*)*, …, a*_*AP*_(*t* + (*d*_*E*_ - 1)τ)] ∈ R^5^
WMaV	5-dimensional delay embedding of V accelerations of L5 (model)	*WMaV*(*t*) *=* [*a*_*V*_(*t*)*, a*_*V*_(*t + τ*)*, …, a*_*V*_(*t* + (*d*_*E*_ - 1)τ)] ∈ R^5^
WMa	Accelerations in the AP and V direction of L5 (model)	*WMa*(*t*) *=* [*a*_*AP*_(*t*)*, a*_*V*_(*t*)] ∈ R^2^
EXPa	Accelerations in the AP and V direction of L5 (experimental)	*EXPa*(*t*) = [*a*_*AP*_(*t*)*, a*_*V*_(*t*)] ∈ R^2^

*φ*_*k,st*_ and *φ*_*k,sw*_ are flexion/extension knee angles for stance and swing limb; similarly, *φ*_*h*,*st*_ and *φ*_*h,sw*_ are flexion/extension hip angles. *φ*_t_ is flexion/extension trunk angle. *a*_*AP*_ and *a*_*V*_ are accelerations of the trunk at the level of L5 in anterior-posterior and vertical directions. For delay-embedded state spaces, τ is time delay and *d*_*E*_ is the embedding dimension (τ = 10, *d*_*E*_ = 5).

For both model and experimental data, a stride cycle was considered as the interval between two consecutive right heel strikes. For experimental data, right heel strike instants were estimated from the angular velocity of the lower limb with a method based on wavelet analysis [33]. Strides were resampled to 101 samples, because Floquet theory requires a strictly periodic system. Experimental data were analysed without filtering, to prevent complications resulting from linear filtering of nonlinear signals [34]. A Poincaré section was defined at each percentage of the gait cycle (0% = right heel strike).

The Poincaré map:

*S*_k+1_ = *F*(*S*_k_) (1)

defines the evolution of the state *S*_*k*_ to the state *S*_*k+1*_ at each Poincaré section, for each stride k.

The limit cycle trajectory was defined as the average trajectory across all strides, defining a fixed point in each Poincaré section:

S^*^ = *F*(S^*^) (2)

A linear approximation of equation (1):

[*S*_k+1_ - S^*^] ^≈^*J*(S^*^)[*S*_k_ - S^*^] (3)

allows to calculate how system states diverge from or converge to fixed points. The FM are the eigenvalues of the Jacobian matrix *J*(S*). The maximum FM (maxFM) is believed to govern the dynamics of the system, and hence to be the most representative in terms of instability. If the maxFM have magnitude < 1, the system is stable, otherwise, the system tends to diverge from the limit cycle and is unstable. maxFM were calculated for each Poincaré section (0 – 100% of the gait cycle), and the overall mean value of maxFM across the gait cycle was calculated and used in this analysis.

Orbital stability analysis on model-data was performed on the seven different state spaces (Table 2). Both noise-free and noisy conditions were analysed, as well as experimental data. Mean values of maxFM along the gait cycle were calculated for increasing number of strides (from 10 to 300 for model-data, from 10 to 160 for experimental data). 

In order to perform a sanity check of the results obtained from numerical calculation of maxFM on model time series, semi-analytical calculation of the FM was performed. The full 10-dimensional state space (composed of angular velocities and accelerations) was considered for this analysis. Instead of estimating S*, the stable period one solution was taken. Ten strides were then simulated (being the state space 10-dimensional), each one with a small perturbation of one the state variables at the heel strike instant. States at heel strike after the perturbation were then put in matrix form; S* was then subtracted from said matrix, obtaining the right hand side of Eq. 3. This matrix was then divided by the magnitude of the initial perturbation in order to obtain *J* matrix [19,35].

## Results

Semi-analytical calculation of the FM of the model led to a value of maxFM_sa_ = 0.23.

**Figure 2:**
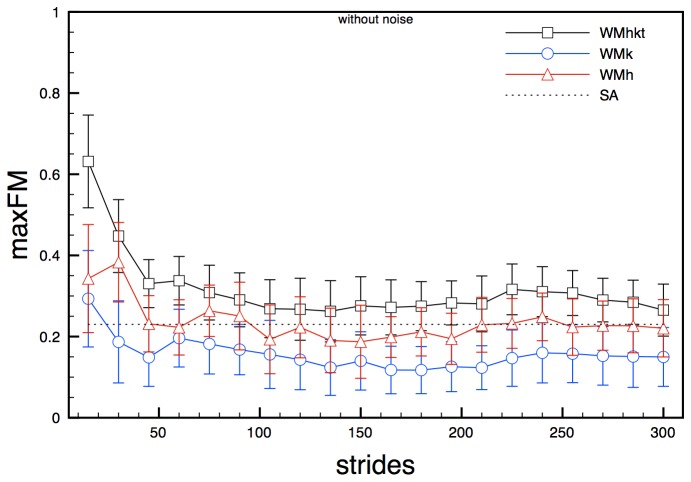
maxFM obtained for model state spaces WMhkt, WMk and WMh (clean signals) for increasing number of stride cycles. Error bars represent standard deviation calculated over the stride cycle. The dotted line (SA) represents the semi-analytical value of the maxFM.

**Figure 3:**
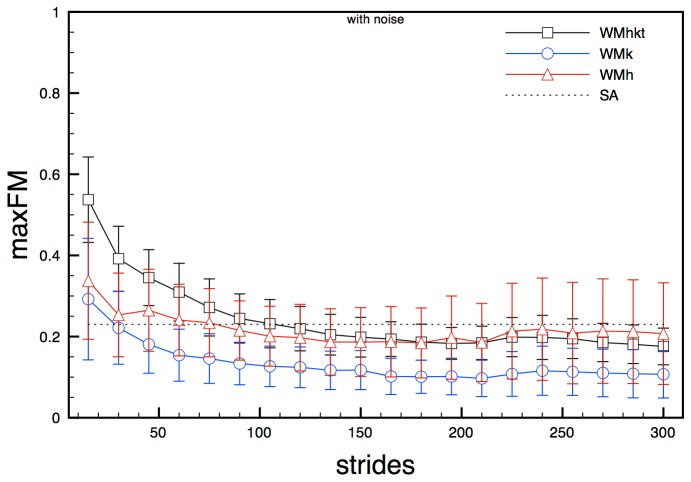
maxFM obtained for model state spaces WMhkt, WMk and WMh (noisy signals) for increasing number of stride cycles. Error bars represent standard deviation calculated over the stride cycle. The dotted line (SA) represents the semi-analytical value of the maxFM.

Experimental noise had a slight but non-negligible influence on maxFM for state spaces composed by joint angles (WMhkt, WMk and WMh). Analysis on these state spaces in noise-free conditions led to mean values of maxFM along the gait cycle that decay with the increase of the analysed stride cycles, until reaching the values 0.27, 0.15 and 0.22 respectively. For all state spaces about 130 strides were needed to reach steady values. Standard deviation slightly decreased with the increase of the number of stride cycles (Figure 2). State spaces composed by noise-affected signals showed a similar overall trend, but seemed to reach slightly different steady values, especially for WMhkt state space (Figure 3).

**Figure 4:**
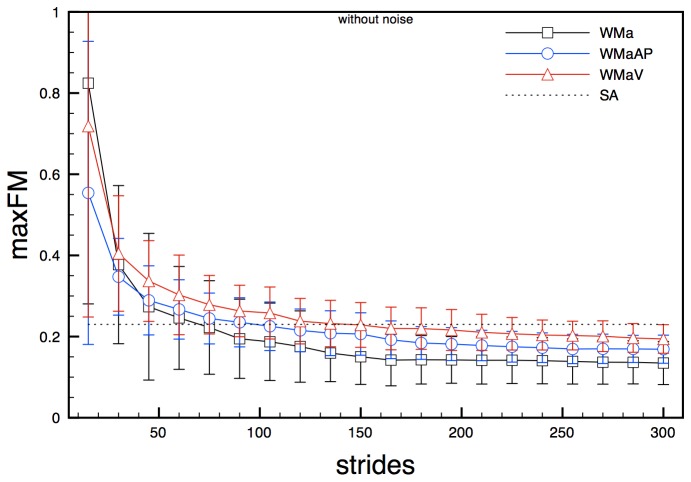
maxFM obtained for model state spaces WMa, WmaAP and WMaV (clean signals) for increasing number of stride cycles. Error bars represent standard deviation calculated over the stride cycle. The dotted line (SA) represents the semi-analytical value of the maxFM.

**Figure 5:**
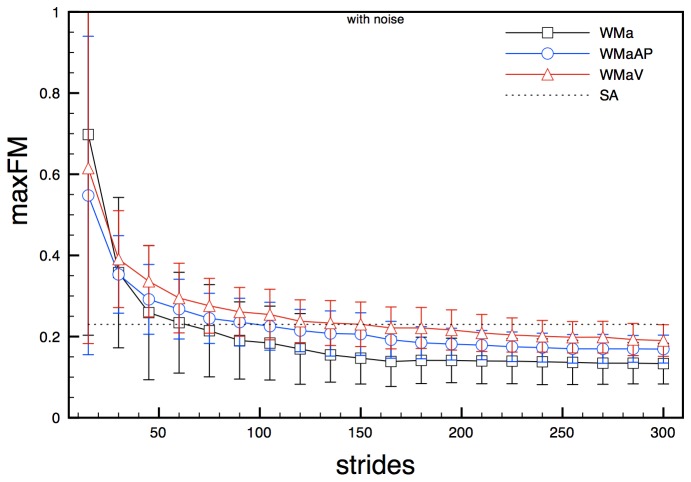
maxFM obtained for model state spaces WMa, WmaAP and WMaV (noisy signals) for increasing number of stride cycles. Error bars represent standard deviation calculated over the stride cycle. The dotted line (SA) represents the semi-analytical value of the maxFM.

MaxFM calculated on noise-free acceleration state spaces, both 2- and 5-dimensional (WMa, WMaAP and WMaV), behaved similarly: values of maxFM gradually decreased, starting from values between 0.5 and 0.9, until stabilizing around values a little lower to the ones previously found for non noise affected joint angle state spaces (0.13 - 0.19) with a standard deviation of about 0.04 (Figure 4). About 130 strides were needed in order to reach steady values. Results coming from analysis of noisy accelerations signals were practically identical to those obtained from noise-free signals for overall trend, number of required strides and numerical values (Figure 5).

**Figure 6:**
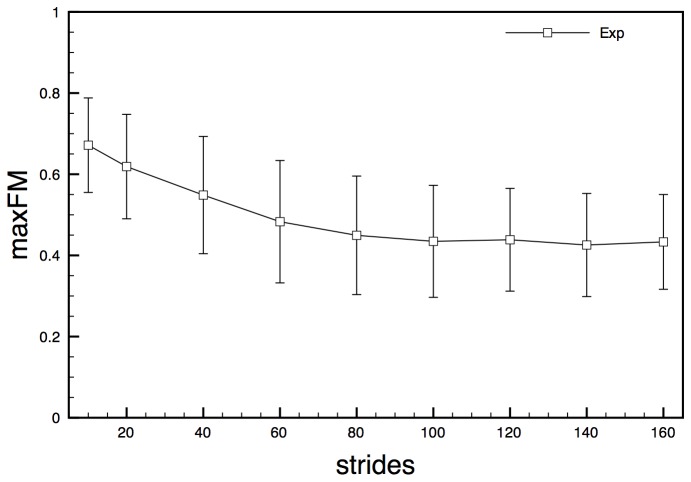
maxFM obtained for experimental state space EXPa for increasing number of stride cycles. Error bars represent standard deviation calculated over the stride cycle.

MaxFM calculated on experimental acceleration state space (EXPa) showed decreasing value for increasing number of cycles analysed, reaching values close to 0.4 from 80 cycles on, with a standard deviation of about 0.1 (Figure 6).

## Discussion

The possibility to have a reliable locomotor stability index is of fundamental importance in early identification and treatment of older adults with high predisposition to fall, and possibly in real-time gait instability detection also. However, still there is no unique definition of locomotor stability in the literature. Orbital stability analysis via maxFM seems promising for the analysis of cyclic locomotor tasks. However, when dealing with biomechanical time series, the equations of motion are unknown, excluding the possibility to calculate maxFM in an analytical or semi-analytical way. Numerical calculation of maxFM from experimental time series is hence required, but it is not clear yet how different implementations of this analysis can influence the stability estimations.

In this explorative study, orbital stability analysis was applied to a 5-link stable walking model. The walking model was used in order to produce signals (joint angles and trunk accelerations) as similar as possible to real human gait signals. Stability was assumed, since the model didn’t show any fall or stumble during the simulation period. Different implementations of numerical orbital stability analysis were then performed on the biomechanical signals obtained from the model. As a reference, semi-analytical calculation of FM of the model was performed. The aim was to better understand the influence of number of analysed cycles, state space composition and experimental noise on the stability outputs.

The magnitude of maxFM obtained in this study was lower than values obtained in previously published simulation studies [19,23,24]. Whereas those studies analysed the behavior of 1- or 2-link walking models, in our study walking of a 5-link model was analysed. A possible explanation is that the higher model complexity allows for a higher number of state variables to compensate for perturbations, thus leading to higher stability. However, as also explicitly stated by Roos and Dingwell [24], the main aim of the previous published works was to show the general relationship between fall risk and stability measures, and not to give exact numerical values.

According to the results of the present study, the number of cycles analysed plays a fundamental role. From a theoretical point of view, the number of analysed cycles cannot be smaller than the dimension of the state space otherwise the set of equations would be underdetermined. Once the dimension of the state space is reached, the analysis of more gait cycles leads to a better estimate of the true attractor [22] in presence of physiological gait variability and experimental noise. 

Orbital stability analysis performed on noise-free signals from the stable walking model resulted in maxFM values close to the reference value of maxFM_sa_ = 0.23, as provided by the semi-analytical calculation of maxFM, for both state spaces composed by joint angles and L5 accelerations. The coherence between these results is encouraging, as it seems to indicate that a repeatable value of the maxFM can be obtained analysing different state spaces. Another similarity among these results was the dependence on the number of analysed cycles, since for all state spaces composed by non noise-affected signals steady results were obtained from about 130 strides on.

For a few number of cycles, maxFM values resulted to be high and inconsistent, hence probably unreliable. Moreover, for shorter time series (15 strides), analysis conducted upon stereophotogrammetric data led to a lower overestimation of the maxFM with respect to the analysis conducted upon acceleration data.

Whereas the analysis performed on 5-dimensional state space WMhkt led to value very close to the semi-analytical value, 2-dimensional state spaces performed comparably, and sometimes slightly better (as it is the case for WMh state space, composed by hip joint angles time series). Whereas a 2-dimensional representation of a complex system may seem insufficient to provide a proper characterization, compared to a 5-dimensional state space, it may serve the applicative purpose of obtaining a repeatable index of stability with a simpler representation of the system dynamics. The relationship with the stability index obtained with this implementation and the actual fall risk remains, however, still undetermined.

Results from the analysis of noisy signals led to slightly different results between acceleration and stereophotogrammetric data. Analysis of noisy accelerations of L5 led basically to the same results obtained for noise-free signals, for all the state spaces: simulated experimental noise on inertial sensor data did not influence maxFM calculation. This can lead to the conclusion that orbital stability analysis performed on state spaces composed by accelerations coming from inertial sensors is robust to noise, and that again a high dimensional (5) reconstruction of the state space may not be necessary, as a lower dimension (2) state space led basically to the same results. Analysis of joint angles showed an influence of experimental noise and mis-localization error, leading to lower steady values for the maxFM, with the exception of WMh which remained practically unvaried (and very close to the reference value of maxFMsa = 0.23).

These results are in agreement with Bruijn et al. [16], who found a correlation of 0.66 between maxFM obtained from two measurement systems (accelerometers and optoelectronics).

Experimental trial results on the accelerations-based state space showed a similar trend with respect to the ones obtained from the analysis of the same variables derived from the model; nevertheless, the value of maxFM obtained was slightly higher, and so the standard deviation. A limitation of this experimental session was the relatively short length of the walks (160 strides) with respect to the model-data; given the high handiness and portability of inertial sensor, however, future studies can analyse orbital stability of very long overground walks. On the other hand, 160 strides seem to be sufficient to reach a steady value for the maxFM.

Based on these results, a reliable implementation of orbital stability analysis could be obtained from an acceleration-based state space (reconstructed with delay-embedding or including in the state space accelerations in different directions) and a number of stride cycles not lower than 130.

In conclusion, the exploration of the influence of experimental input parameters in orbital stability analysis led to interesting results. One of the main issues relative to this technique is the necessity to properly describe the dynamical system, in order to obtain a reliable orbital stability index; hence, the definition of the state space is of crucial importance for the outputs. The coherence between the results obtained with differently composed state spaces showed that the same stability output can be obtained with different implementations and experimental setup. The number of gait cycles necessary to obtain these results is also practically identical among these setups. For the peculiarity of the instrumentation features, however, stereophotogrammetry system is only suitable for acquiring such long gait trials when a treadmill is used.

Experimental noise and operator errors have an impact, although small, on the results when using orbital stability analysis based on joint angles obtained from stereophotogrammetric systems. Further studies are needed to determine if the stability measures obtained from analysis on these state spaces are really capable to discriminate between known stability conditions. Experimental noise on accelerometer data showed no particular influence on the stability results.

Experimental results were also coherent with the model results in terms of number of cycles required, supporting the validity of the stability outcomes. This result confirms the possibility to obtain reliable orbital stability measures with a single inertial sensor and could lead to advantages in the development of a simple and fast data acquisition protocol, confirming what was found in literature for treadmill walking [16].
